# Participation in a Diabetes Self-Management Class Among Adults With Diabetes, New Jersey 2013–2015

**DOI:** 10.5888/pcd14.170023

**Published:** 2017-08-03

**Authors:** Melissa L. Santorelli, Ruwani M. Ekanayake, LorieAnn Wilkerson-Leconte

**Affiliations:** 1 Community Health and Wellness Unit, New Jersey Department of Health, Trenton, New Jersey

## Abstract

Identifying patient groups with low participation in diabetes self-management education can inform efforts to improve its use. Data from the 2013–2015 Behavioral Risk Factor Surveillance System were used to assess variation in participation in a diabetes self-management class in New Jersey. Nonparticipation varied significantly by race/ethnicity (*P* < .001), education (*P* < .001), health care coverage (*P* = .04), county (*P* < .001), years since diagnosis (*P* < .001), and whether a diabetes provider visit occurred in the past year (*P* = .002). Attention is warranted in identifying participation barriers among patients who live in certain counties, have less education, are without health care coverage, have been diagnosed with diabetes more recently, visit a provider less often, or belong to certain racial/ethnic minority groups.

## Objectives

Diabetes self-management education (DSME) is defined as “the process of facilitating the knowledge, skill, and ability necessary for diabetes self-care” (1). Research has linked DSME with improved glycemic control and various indicators of preventive care (1,2). Identifying patients with low participation can inform efforts to promote DSME; however, few studies have examined the characteristics of nonparticipants (2,3). These efforts are important in New Jersey where participation falls below that of many other states (4). Therefore, we evaluated variation in DSME nonparticipation by various factors. We also evaluated whether a geographic association exists between program availability and program need.

## Methods

The Behavioral Risk Factor Surveillance System (BRFSS) is a state-based landline and cellular telephone survey of noninstitutionalized, civilian adults in the United States. A cross-sectional study was performed using New Jersey data from the 2013–2015 BRFSS. Survey respondents were asked whether a doctor, nurse, or other health professional had ever told them they had diabetes. Respondents who reported only a history of gestational diabetes or prediabetes were excluded, resulting in a total of 4,397 respondents with diabetes. These respondents were asked, “Have you ever taken a course or class in how to manage your diabetes yourself?” Those who did not answer (n = 39) were excluded, leaving a final sample of 4,358. Research on the reliability and validity of BRFSS questions has been published (5).

We used SAS version 9.2 complex survey procedures (SAS Institute, Inc). Before combining the annual samples, we used the Rao-Scott χ^2^ test to confirm that annual estimates for diabetes prevalence and the percentage of New Jersey adults with diabetes who never participated in a diabetes self-management class were similar. After combining the annual samples, annual weights were adjusted on the basis of contribution to the overall sample. Diabetes prevalence and class nonparticipation were estimated overall and by county. Nonparticipation was also estimated by various demographic, socioeconomic, and clinical factors. The Rao-Scott χ^2^ test was used to assess the bivariate association between each factor and nonparticipation. We used ArcGIS 10.3.1 (Esri) to geographically display nonparticipation by county, using the Jenks natural breaks classification method (6). We also calculated the number of diabetes self-management programs per 100,000 adults with diabetes by county and overlaid graduated symbols reflecting these rates. The New Jersey Diabetes Prevention and Control Program maintains a statewide listing of diabetes self-management programs (recognized by the American Diabetes Association [ADA], accredited by the American Association of Diabetes Educators [AADE], or licensed by Stanford University); this listing was used to identify the number of programs in each county.

## Results

The overall New Jersey annual BRFSS response rates were 41.4% (2013), 47.5% (2014), and 46.6% (2015) (7–9). The estimates for diabetes prevalence (*P* = .20) and for class nonparticipation (*P* = .28) did not vary significantly by year. Based on the combined 3-year sample, the total number of New Jersey adults with diabetes was estimated to be 643,817 (9.3%; 95% confidence interval [CI], 8.9%–9.7%); 58.0% (95% CI, 55.8%–60.3%) of these adults never participated in a diabetes self-management class. Estimated class nonparticipation varied significantly by race/ethnicity (*P* < .001), education (*P* < .001), health care coverage (*P* = .04), years since diagnosis (*P* < .001), and whether a provider visit for diabetes occurred in the past year (*P* =.002) (Table).

**Table:** Association Between Demographic, Socioeconomic, and Clinical Factors and Nonparticipation in a Diabetes Self-Management Class Among New Jersey Adults With Diabetes, Behavioral Risk Factor Surveillance System, 2013–2015

Characteristic	Never Participated in Diabetes Self-Management Class, % (95% Confidence Interval)	*P* Value^b^
**Age group, y (n = 4,358)^a^**		
18–24	— c	.32
25–34	56.0 (39.3–72.6)	
35–44	49.5 (40.0–59.0)	
45–54	55.6 (49.9–61.2)	
55–64	57.1 (52.7–61.5)	
≥65	60.8 (57.7–63.9)	
**Sex (n = 4,358)**		
Male	58.4 (55.1–61.7)	.76
Female	57.7 (54.7–60.7)	
**Race/ethnicity (n = 4,236)**		
White, non-Hispanic	54.7 (51.9–57.5)	<.001
Black, non-Hispanic	54.2 (48.4–60.0)	
Multiracial, non-Hispanic	— c	
Other, non-Hispanic^d^	72.9 (64.0–81.7)	
Hispanic	65.2 (59.7–70.8)	
**Household income, $ (n = 4,358)**		
<15,000	57.9 (50.6–65.2)	.17
15,000 to <25,000	61.4 (56.4–66.5)	
25,000 to <35,000	55.9 (48.7–63.1)	
35,000 to <50,000	62.2 (55.3–69.2)	
≥50,000	54.5 (50.6–58.3)	
Not reported	60.2 (54.9–65.4)	
**Education (n = 4,303)**		
Less than a high school diploma	66.8 (60.7–72.9)	<.001
High school graduate	60.4 (56.6–64.2)	
Some college/technical school	49.7 (45.3–54.2)	
Graduated college/technical school	55.6 (51.3–59.9)	
**Health care coverage (n = 4,342)**		
Yes	57.4 (55.1–59.8)	.04
No	66.3 (58.3–74.3)	
**Years since diagnosis (n = 4,003)**		
0 to less than 2	70.3 (63.4–77.2)	<.001
2 to less than 5	62.1 (56.3–67.9)	
5 to less than 10	59.4 (54.5–64.4)	
10 or more	52.7 (49.4–56.0)	
**Provider visit for diabetes in past year (n = 4,149)**		
Yes	56.7 (54.3–59.2)	.002
No	68.0 (61.6–74.5)	
**All adults (n = 4,358)**	58.0 (55.8–60.3)	NA

Abbreviation: NA, not applicable.

a Sample sizes vary because of missing values.

b Rao-Scott χ^2^ test was used to assess the association between each factor and class nonparticipation.

c Estimate unreliable because of small sample size.

d Includes American Indian/Alaska Native, Asian, Native Hawaiian/Pacific Islander, and other race/ethnicity.

The estimated percentage of adults with diabetes who never participated in a diabetes self-management class and the number of diabetes self-management programs per 100,000 adults with diabetes are shown by New Jersey county in the Figure. Class nonparticipation varied by county (*P* < .001), ranging from 41.5% (95% CI, 30.3–52.6) of adults with diabetes in Somerset County to 69.8% (95% CI, 62.6–76.9) of adults with diabetes in Cumberland County. Program availability ranged from 3.6 programs per 100,000 residents with diabetes in Morris County to 31.9 programs per 100,000 residents with diabetes in Salem County.

**Figure:**
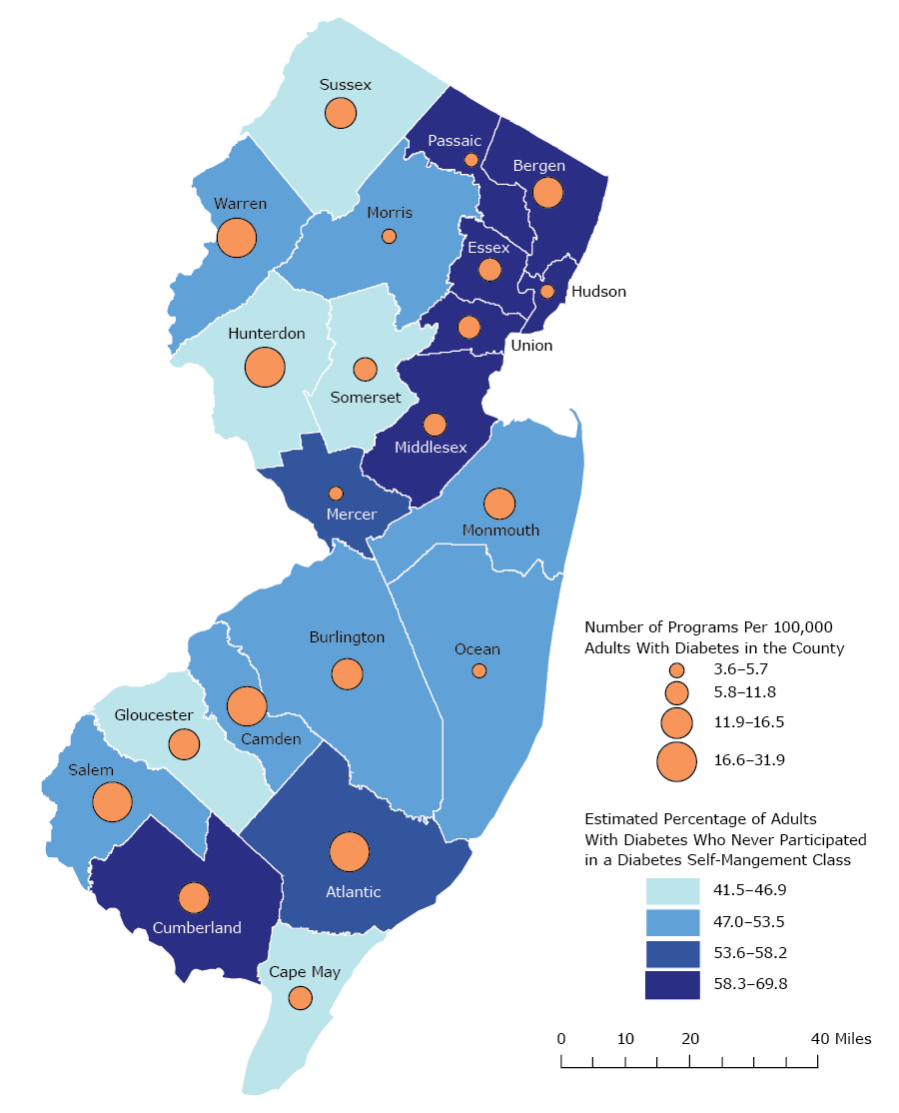
Estimated percentage of New Jersey adults with diabetes who have never participated in a diabetes self-management class (Behavioral Risk Factor Surveillance System [BRFSS] 2013–2015), and number of diabetes self-management programs (New Jersey Diabetes Prevention and Control Program) per 100,000 adults with diabetes (BRFSS 2013–2015), by New Jersey county. **Table:** 

County	Number of Programs Per 100,000 Adults With Diabetes	Estimated Percentage of Adults With Diabetes Who Never Participated in a Diabetes Self-Management Class
Ocean	3.6–5.7	47.0–53.5
Mercer	3.6–5.7	53.6–58.2
Morris	3.6–5.7	47.0–53.5
Hudson	3.6–5.7	58.3–69.8
Passaic	3.6–5.7	58.3–69.8
Cape May	5.8–11.8	41.5–46.9
Middlesex	5.8–11.8	58.3–69.8
Somerset	5.8–11.8	41.5–46.9
Union	5.8–11.8	58.3–69.8
Essex	5.8–11.8	58.3–69.8
Gloucester	11.9–16.5	41.5–46.9
Cumberland	11.9–16.5	58.3–69.8
Burlington	11.9–16.5	47.0–53.5
Monmouth	11.9–16.5	47.0–53.5
Bergen	11.9–16.5	58.3–69.8
Sussex	11.9–16.5	41.5–46.9
Atlantic	16.6–31.9	53.6–58.2
Salem	16.6–31.9	47.0–53.5
Camden	16.6–31.9	47.0–53.5
Hunterdon	16.6–31.9	41.5–46.9
Warren	16.6–31.9	47.0–53.5

## Discussion

Our findings suggest that efforts to promote DSME should target participation barriers among patients who live in certain counties, have less education, who are without health care coverage, were diagnosed recently, visit a diabetes provider less often, or who identify as Hispanic or non-Hispanic other race (American Indian/Alaska Native, Asian, Native Hawaiian/Pacific Islander, other). These results are consistent with those of previous studies showing that nonparticipants were more likely to belong to minority racial/ethnic groups and have less education (2,3). Our findings also suggest that lower participation in certain areas may not always reflect program availability. In some counties where the need was highest, the number of programs was lowest (Passaic, Hudson); however, this association was not apparent in other counties with a high level of need (Bergen, Cumberland).

This study has several limitations. Respondents may have attended a course or class that was not ADA-recognized, AADE-accredited, or Stanford University–licensed; such programs were not considered in the geographic analysis. This analysis was conducted at the county level; therefore, any association between program availability and nonparticipation that exists below this level would not have been detected. Finally, we considered only the number of programs as a measure of program availability; other factors such as geographical reach, cultural or linguistic capacity, operating hours, influence of strategic partnerships, and venue characteristics may be important. This study also has several strengths. The results represent an estimated 643,817 New Jersey adults with diabetes. Also, the findings and methods may have much broader relevance beyond New Jersey, because promoting DSME continues to be a national public health priority (10).

The issue of DSME nonparticipation is complex. Community needs assessments should consider how patient-level and program-level factors contribute to nonparticipation among residents. Study findings can be used to focus these efforts on patient groups that exhibit low use of DMSE programs.
